# Repurposing ritonavir to induce proteasomal degradation of IE1 for inhibition of human cytomegalovirus replication

**DOI:** 10.1371/journal.pcbi.1014867

**Published:** 2026-10-06

**Authors:** Wanfeng Liu, Yu Shi, Xudong Guo, Chenyu Lou, Zhangyi Xie, Yitian Shen, Liang Zhang, Sunzhong Mao, Xiangyang Xue, Shiyu Feng

**Affiliations:** 1 Wenzhou Collaborative Innovation Center of Gastrointestinal Cancer in Basic Research and Precision Medicine, Wenzhou Key Laboratory of Cancer-related Pathogens and Immunity, Department of Microbiology and Immunology, School of Basic Medical Sciences, Wenzhou Medical University, Wenzhou, China; 2 College of Information Engineering, Northwest A&F University, Xianyang, Shaanxi, China; 3 The Second Affiliated Hospital, Yuying Children’s Hospital of Wenzhou Medical University, Wenzhou, China; 4 Zhejiang Key Laboratory of Intelligent Cancer Biomarker Discovery and Translation, The First Affiliated Hospital of Wenzhou Medical University, Wenzhou, Zhejiang, China; University of California San Diego Division of Biological Sciences, UNITED STATES OF AMERICA

## Abstract

Human cytomegalovirus (HCMV) is a major pathogen affecting immunocompromised individuals. Current antiviral drugs target the mid-to-late stages of viral replication but are limited by toxicity and drug resistance. The IE1 protein serves as a key early regulator of HCMV replication; however, it has long been considered undruggable due to the lack of conventional drug-binding sites. In this study, by integrating molecular docking-based virtual screening with *in-vitro* surface plasmon resonance (SPR) validation, we discovered for the first time that the HIV protease inhibitor ritonavir may bind to the IE1 protein. Further experiments demonstrated that ritonavir significantly reduces IE1 protein levels in a time- and dose-dependent manner without affecting its mRNA expression. Mechanistic investigations revealed that ritonavir promotes selective degradation of IE1 through activation of the ubiquitin-proteasome pathway. Functional assays confirmed that ritonavir effectively inhibits HCMV replication, markedly reducing viral DNA loads and the production of infectious viral particles. Notably, ritonavir exhibited synergistic antiviral effects when combined with the first-line drug ganciclovir (GCV). In summary, this study successfully repurposes the approved drug ritonavir as a regulator of IE1 protein expression, demonstrating not only its anti-HCMV activity and synergistic effects with GCV but also providing a new therapeutic strategy for HCMV infection.

## Author summary

Human cytomegalovirus (HCMV) represents a major threat to immunocompromised patients. However, currently available antivirals, which primarily target the mid-to-late stages of viral replication, are limited by toxicity, drug resistance, and insufficient efficacy against latent infection. Early viral regulators therefore constitute promising therapeutic targets. Here, using molecular docking-guided drug repurposing, followed by *in vitro* SPR binding assays we identified the HIV protease inhibitor ritonavir as a binder of the immediate-early protein IE1, a critical early regulator of HCMV replication that has long been considered undruggable owing to the absence of canonical drug-binding pockets. Ritonavir promoted ubiquitin–proteasome-dependent degradation of IE1, thereby exerting antiviral activity. Notably, ritonavir exhibited synergistic antiviral effects when combined with GCV. These findings provide a mechanistic rationale for combining drug-repurposing strategies with proteasome-dependent degradation approaches in future anti-HCMV investigations.

## Introduction

Human cytomegalovirus (HCMV), a prevalent member of the *Betaherpesvirinae* subfamily [1], is a major opportunistic pathogen causing severe diseases such as retinitis [2], interstitial pneumonia [3], and neurological impairment [4], particularly in immunocompromised individuals including neonates [5], transplant recipients [6], and patients with HIV infection [2]. Current antivirals, such as GCV [7], which inhibits viral DNA polymerase, and letermovir (LMV) [8], which targets the terminase complex, act mainly during the mid to late stages of viral replication. Although effective, their long term use is substantially limited by hematologic toxicity, emergence of resistance, and poor activity against latent viral reservoirs [9–11]. Therefore, the development of novel therapeutic strategies that act on new targets and, in particular, can overcome drug resistance is of critical importance.

The immediate-early (IE) protein serves as the central regulator for initiating lytic replication in human cytomegalovirus (HCMV) [12,13]. Through alternative splicing of its transcript, this protein generates two major functional isoforms—IE1 (pUL123) and IE2 (pUL122)—which play overlapping yet complementary critical roles in the viral life cycle [14]. As a key regulator of viral gene expression, the IE protein coordinates viral replication and remodels the host cellular environment at multiple levels. At the transcriptional level, the IE protein cooperatively activates the cascade expression of early and late viral genes, ensuring the orderly progression of viral gene transcription [15–17]. At the translational level, the IE protein inhibits host translational repressors (such as PKR), thereby guaranteeing efficient viral protein synthesis and promoting the preferential translation of viral mRNAs [18]. In terms of immune evasion, IE1 interferes with the host STAT1/STAT2 signaling pathway, suppresses type I interferon-mediated antiviral responses, and thus helps the virus evade innate immune surveillance [19,20]. Furthermore, IE1 modulates the host cell cycle, for example by promoting the transition of cells from the G1 to S phase, thereby creating an intracellular environment favorable for viral replication [21,22]. In summary, the IE protein drives viral replication and suppresses host defenses through multiple mechanisms, including transcriptional regulation, translational control, immune evasion, and cell cycle modulation.

Several studies have confirmed that targeting the expression or function of the IE1 protein—whether through antisense oligonucleotides, RNA interference, gene editing, or small-molecule inhibitors—effectively blocks HCMV replication [23,24], establishing the theoretical foundation for the IE protein as a crucial antiviral target. However, drug development targeting IE1 faces unique structural challenges: unlike many classical enzymatic targets, IE1 lacks a well-defined catalytic pocket or typical active site [25], making traditional structure-based small-molecule inhibitor design strategies difficult to apply directly. Consequently, it has long been considered undruggable.

In this study, we employed molecular docking-based virtual screening and, using SPR assays,verified that the HIV protease inhibitor ritonavir interacts directly with the IE1 protein, specifically down-regulate IE1 protein abundance in a time- and dose-dependent manner and subsequent suppression of viral replication. Further mechanistic studies confirmed that ritonavir promotes IE1 degradation primarily through the ubiquitin-proteasome pathway. Based on these findings, we demonstrated that the combination of ritonavir with GCV exerts a significant synergistic antiviral effect, enhancing antiviral activity. In summary, this study not only reveals *in vitro* the therapeutic potential of ritonavir as an anti-HCMV agent but also proposes a novel antiviral strategy based on proteasome-dependent degradation of a key immediate-early viral protein.

## Materials and methods

Ethics statement: This study did not involve human participants, human data, or animals, and therefore ethical approval was not required according to the guidelines of the journal.

### Molecular docking-based virtual screening

The resolved crystal structure of the IE1 central core domain (PDB ID: 6TGZ) was used as the receptor model [25,26]. Protein side-chain protonation was performed at pH 7.0, and standard charges were assigned to the protein N and C-termini for structural preprocessing. A total of 890 small-molecule compounds from the FDA-approved drug library were subjected to high-throughput molecular docking using Discovery Studio 2019 software [27]. Prior to docking, the ligands were subjected to hydrogen addition, conformational sampling, and stereoisomer optimization. Docking was performed using the LibDock algorithm, and compounds were ranked based on their LibDock scores. Those with scores exceeding 150 were selected as candidate molecules.

To further evaluate the potential binding propensity of the candidate compounds, molecular docking was performed using AutoDock-GPU v2. Briefly, CB-Dock2 was first applied for blind global surface scanning of IE1 to identify putative binding pockets and define the primary binding region. Subsequent site-specific docking was conducted with the preprocessed IE1 structure treated as a rigid receptor and each candidate hit defined as a flexible ligand. The docking grid was centered at (11.010, -15.048, 0.547) with dimensions of 27 × 27 × 35 Å, fully enclosing the predicted pocket. The optimal binding pose was selected based on computed binding free energy. Notably, docking scores in this work were used only for relative ranking of ligand-binding potency within identical simulation systems to prioritize promising IE1-targeting candidates. They cannot reflect absolute binding affinity or quantitatively reproduce experimentally measured affinity values [28]. In this site-specific docking calculation, the IE1 receptor was kept rigid while all rotatable bonds of each ligand were set flexible. A grid spacing of 0.375 Å was applied. The Lamarckian genetic algorithm parameters were inherited from the CB-Dock2 output configuration file: population size 150, maximum energy evaluations 2 500 000, 27 000 generations.

### Molecular dynamics

The top-ranked IE1–ritonavir binding pose with the most favorable predicted docking energy from AutoDock was selected as the initial structure for molecular dynamics (MD) simulations. Both apo-IE1 and the IE1–ritonavir complex were subjected to MD simulations under identical conditions. All MD simulations were performed on the WeMol platform (https://wemol.wecomput.com) using the built-in GROMACS 2024 workflow for explicit solvent calculations. The amber14sb_parmbsc1 force field was assigned for the protein, and the TIP3P water model was employed. Ritonavir was hydrogenated and fully parameterized prior to simulation. The complex was placed in a cubic periodic box with a minimum distance of 1.0 nm between protein atoms and box walls, and ionic neutralization was performed to neutralize residual charge. Energy minimization was carried out via the steepest descent algorithm with a convergence threshold of 100 kJ/(mol·nm). Subsequently, a 10 ns NPT equilibration was conducted at 300 K and 1 bar with the protein main chain position restrained. Temperature was controlled via a V-rescale thermostat, and pressure coupling was performed using the Parrinello-Rahman barostat. Long-range electrostatics were computed using the PME method, with a non-bonded cutoff of 1.0 nm. Hydrogen bonds were constrained, and the integration step was 1 fs. After equilibration, restraints were released, and a 100 ns production run was initiated with a 2 fs step and a 1.2 nm cutoff. Trajectories were output every 10 ps. Based on the MD trajectories, root-mean-square deviation (RMSD), radius of gyration (Rg), root-mean-square fluctuation (RMSF), hydrogen-bond counts, solvent-accessible surface area (SASA), and secondary-structure changes were analyzed. Principal component analysis (PCA) was applied to construct the free-energy landscape. For MM-PBSA calculations, the 50–100 ns trajectory segment was extracted to ensure equilibration, and binding free energies along with residue-level contributions were determined. Simulation convergence was judged by a stable plateau of backbone RMSD and Rg.

### Surface plasmon resonance (SPR)

SPR experiments were performed using the Biacore 1K system equipped with a CM5 sensor chip to assess the direct physical interaction between ritonavir and IE1 protein *in vitro*. The recombinant IE1 protein was immobilized via the amine-coupling method, using 1 × PBS-P+ (pH 7.4) as the immobilization running buffer. The interaction running buffer consisted of 1 × PBS-P+ (pH 7.4) supplemented with 1% (v/v) DMSO. The flow rate was maintained at 10 μL/min throughout the experiment.

The chip surface was activated for 420 seconds with an equal-volume mixture of 0.4 M EDC and 0.1 M NHS. IE1 protein was diluted to 50 μg/mL in 10 mM sodium acetate buffer (pH 4.5) and injected into the detection channel (channel 2) for covalent immobilization. The blank reference channel (channel 1) was treated with sodium acetate buffer alone without protein. Both channels were then blocked with 1 M ethanolamine hydrochloride (pH 8.5) for 420 seconds to quench residual active sites on the chip surface.

Ritonavir was serially diluted to concentrations ranging from 12.5 nM to 6400 nM and injected sequentially for kinetic measurements. The association and dissociation phases were set to 100 s and 120 s, respectively. After each concentration cycle, the chip was regenerated with 10 mM glycine-HCl (pH 2.0) for 5 min to restore binding capacity. Following subtraction of the reference channel signal, the resulting sensorgrams were globally fitted using a 1:1 Langmuir binding model to determine the association rate constant (*k*_*a*_), dissociation rate constant (*k*_*d*_), and equilibrium dissociation constant (*K*_*D*_).

### Cells and viruses

Human foreskin fibroblasts (HFF) and HeLa cells were obtained from the American Type Culture Collection (ATCC, Manassas, VA, USA). The identity of all cell lines was authenticated by short tandem repeat (STR) analysis and confirmed to be free of mycoplasma contamination prior to use. Cells were cultured in Dulbecco’s modified Eagle’s medium (DMEM; Gibco, Thermo Fisher Scientific, Waltham, MA, USA) supplemented with 10% fetal bovine serum (FBS; Gibco) and 1% penicillin-streptomycin (Gibco). Cultures were maintained at 37°C in a humidified incubator with 5% CO_2_.

Two HCMV strains were used in this study: the laboratory-adapted strain AD169 (ATCC) and a recombinant HCMV expressing GFP (rHCMV), which was kindly provided by Prof. Minhua Luo (Wuhan Institute of Virology, Chinese Academy of Sciences, Wuhan, China). Viral stocks were propagated in confluent HFF monolayers until complete cytopathic effects were observed. The culture supernatants were clarified by low-speed centrifugation, and virions were purified by sucrose density gradient ultracentrifugation. Purified viruses were aliquoted and stored at -80°C until use. Viral titers were determined using the 50% tissue culture infectious dose (TCID_50_) assay on HFF cells.

### Cell viability assay

HFF cell viability after ritonavir treatment was determined using a CCK-8 kit (YEASEN, Shanghai, China). Briefly, 1 × 10^4^ cells were seeded in 96-well plates and incubated overnight. Cells were treated with 5 μM ritonavir or an equal volume of DMSO (control) for 5 days. After washing with PBS, culture medium containing 10% CCK-8 reagent was added, followed by incubation for 1 h at 37°C. Absorbance at 450 nm was measured using a Varioskan LUX microplate reader (Thermo Fisher Scientific). Cell viability was expressed as a percentage relative to the DMSO control.

### Apoptosis assay

Apoptosis was assessed using an Annexin V–FITC/PI Apoptosis Detection Kit (YEASEN) and flow cytometry. HFF cells were treated with 5 μM ritonavir for 5 days, collected with trypsin (without EDTA), washed with PBS, and resuspended in 100 μL of 1 × Binding Buffer (1 × 10^5^ cells). Cells were stained with 5 μL Annexin V–FITC and 10 μL PI for 10 min at room temperature in the dark, followed by addition of 400 μL Binding Buffer. Samples were analyzed within 1 h using a flow cytometer (BD Biosciences, San Jose, CA, USA). The apoptosis rate was calculated as the sum of early and late apoptotic cells.

### Viral DNA extraction and quantitative PCR

Ritonavir antiviral activity was evaluated by quantifying intracellular and extracellular HCMV DNA. After 5 days of treatment, AD169-infected HFF cells and culture supernatants were collected separately. Total DNA was extracted from the cells, whereas viral DNA was extracted from the culture supernatants using commercial DNA extraction kits (YEASEN) according to the manufacturer’s instructions. A standard curve for the IE1 gene was generated by serial dilution of a pcDNA3.1-IE1 plasmid of known concentration. Viral DNA copy numbers were calculated from sample Ct values using this standard curve. Viral DNA loads from cellular lysates and culture supernatants were quantified using qPCR. Intracellular viral DNA was normalized to total cellular DNA (HCMV copies per ng total cellular DNA), and supernatant-derived extracellular viral DNA was presented as HCMV copies per mL culture supernatant.

### Determination of EC_50_, CC_50_ and SI

HFF cells were infected with AD169 and treated with escalating concentrations of ritonavir/LMV/GCV for 5 days. The cells were then harvested, and total cellular DNA was extracted, Intracellular HCMV DNA was quantified by qPCR and normalized to total cellular DNA. Antiviral inhibition rates were calculated relative to the untreated infected control, and dose–response curves were fitted by nonlinear regression to determine the half-maximal effective concentration (EC_50_). Meanwhile, cell viability was assessed using a gradient concentration CCK-8 assay, and the half-maximal cytotoxic concentration (CC_50_) was obtained through curve fitting. The selectivity index (SI) was calculated as CC_50_/EC_50_ to evaluate the drug’s safety margin *in vitro.*

### Virus titer assay

Antiviral activity was determined by TCID_50_ assay [29]. HFF were cultured in logarithmic phase and seeded into 96-well plates. Cells were infected with HCMV and treated with either single agents or drug combinations for 5 days. The treatment groups included ritonavir (5 μM), GCV (1 or 2 μM), ritonavir/GCV combinations (5 μM/2 μM and 5 μM/1 μM), LMV (2 or 6 nM), and ritonavir/LMV combinations (5 μM/2 nM and 5 μM/6 nM). Untreated infected cells served as controls. Following the 5-day treatment period, culture supernatants were collected for viral titration. Log-phase HFF cells were reseeded into 96-well plates and inoculated with serial 10-fold dilutions of each supernatant in eight replicate wells. Uninfected cells were used as negative controls. The plates were incubated at 37°C in a 5% CO_2_ atmosphere for 7 days, during which cytopathic effects (CPE) were recorded daily. TCID_50_ values were calculated using the Reed–Muench method and subsequently converted to plaque-forming units (PFU) according to the formula: estimated PFU ≈ 0.7 × TCID_50_. Viral titers were expressed as PFU/mL. This 0.7 is merely the theoretical conversion coefficient for the unit titration volume of the same volume; it does not include the dilution factor and the correction for the inoculation volume; all dilutions and volume conversions have been completed during the TCID_50_ titration calculation stage [30]. To evaluate the antiviral efficacy of single agents and combinations, the viral inhibition rate was calculated for each treatment condition. Single-agent and combination viral inhibition rates were derived from *I=(1 − T/C) × 100%*, where T and C denote viral loads of treated and untreated groups, respectively. The expected additive inhibition rates for drug combinations were calculated based on the Bliss independence model. Based on the Bliss model, the theoretical additive inhibition was calculated using the formula: *E*_*AB*_ = *E*_*A*_ + *E*_*B*_ *- E*_*A*_*E*_*B*_*,* with E_*A*_ and E_*B*_ representing the individual inhibition rates of each drug at the corresponding concentrations, *E*_*AB*_ is the theoretical additive inhibition predicted by the Bliss independence model. All Bliss-model synergy analyses were performed via the free online web-tool SynergyFinder (https://synergyfinder.fimm.fi). According to SynergyFinder documentation, a synergy score >10 indicates synergistic interaction; scores between −10 and 10 indicate additive effects; a synergy score <−10 indicates antagonism. Detailed data can be found in S4 Table.

### Plasmid transfection

HeLa cells were seeded in 12-well culture plates and grown to 70–80% confluence. Different overexpression plasmids encoding HA-tagged target genes and the Flag-IE1-expressing plasmid were separately transiently transfected into cells using Lipofectamine 2000 transfection reagent (Thermo Fisher Scientific, USA) according to the manufacturer’s instructions. The culture medium was replaced with fresh complete medium 4–6 h post-transfection. 24 hours after transfection, cells were treated with different concentrations of ritonavir. Cell samples were harvested five days post-treatment.

### Immunofluorescence assay

HFF cells were cultured on glass coverslips in 12-well plates. After viral infection and ritonavir treatment for 5 days, cells were fixed with 4% paraformaldehyde for 15 min and permeabilized with 0.5% Triton X-100 on ice for 10 min. After blocking with 5% goat serum in 5% BSA for 1 h at room temperature, cells were incubated with mouse anti-IE1 antibody (Abcam, Cambridge, UK) overnight at 4°C, followed by Alexa Fluor 594-conjugated goat anti-mouse IgG (Invitrogen, Thermo Fisher Scientific, Waltham, MA, USA) for 1 h at room temperature. Nuclei were stained with DAPI. Images were acquired using a Leica STELLARIS 5 confocal microscope, and IE1 fluorescence intensity was quantified using ImageJ software.

### Western blot

HFF or IE1 stable expression cells were collected after the indicated treatments and lysed in RIPA buffer (Beyotime, Shanghai, China) containing protease and phosphatase inhibitors (MCE). Protein concentrations were determined using a BCA assay kit (Beyotime). Equal amounts of protein were separated by 10% SDS-PAGE, transferred to PVDF membranes, and blocked with 5% non-fat milk for 1 h at room temperature. Membranes were incubated overnight at 4°C with primary antibodies against IE1, UL44 (Virusys Corporation, Taneytown, MD, USA), UL83 (Santa Cruz Biotechnology, Dallas, TX, USA), UL84 (Santa Cruz Biotechnology), GAPDH, β-actin, and α-tubulin (Huabio, Hangzhou, China); HSP90 and HA (Proteintech, Wuhan, Hubei, China); and ubiquitin (Cell Signaling Technology), followed by HRP-conjugated secondary antibodies (Cell Signaling Technology, Danvers, MA, USA) for 1 h at room temperature. Signals were detected using an ECL substrate (Thermo Fisher Scientific) and quantified with ImageJ software, normalized to GAPDH. Uncropped, full-length original western blot membranes for all immunoblot results are available in the annotated file S1_raw_images hosted at Zenodo (10.5281/zenodo.22170116).

### RNA extraction and RT-qPCR

HFF cells infected with the AD169 strain were treated with ritonavir for 1, 2, 3, 5 days. Total RNA was extracted using TRIzol reagent (Thermo Fisher Scientific) and reverse-transcribed into cDNA with the HiScript III 1st Strand cDNA Synthesis Kit (Vazyme, Nanjing, China). RT-qPCR was performed with SYBR Premix Ex Taq II (YEASEN). β-actin was used as the internal control, and relative expression levels were calculated using the 2^−ΔΔCt^ method. The primer sequences used in this study were as follows:

*β-actin* forward: 5′-CTCTTCCAGCCTTCCTTCCT-3′;
*β-actin* reverse: 5′-AGCACTGTGTTGGCGTACAG-3′;
*IE1* forward: 5′-GAGACACCCGTGACCAAGGC-3′;
*IE1* reverse: 5′-CGCACCATGTCCACTCGAAC-3′;
*UL44* forward: 5′-CAGCTGCACGTTGATACGCATGT-3′;
*UL44* reverse: 5′-TCCACCGGCCATCAAGTTTATCCT-3′.

### Co-immunoprecipitation

HeLa cells stably expressing Flag-tagged IE1 were treated with ritonavir for 5 days. Whole-cell lysates were prepared using NP-40 lysis buffer (Beyotime) supplemented with protease inhibitors. Flag-tagged IE1 was immunoprecipitated with 2 μg of anti-Flag antibody (Sigma-Aldrich), with species-matched control IgG used as a negative control. After overnight incubation at 4°C, Protein A/G magnetic beads (Beyotime) were added and incubated for an additional 3 h. The beads were washed and eluted by boiling in 2 × SDS loading buffer. Ubiquitin signals associated with immunoprecipitated Flag-IE1 were detected by Western blotting using an anti-ubiquitin antibody (Cell Signaling Technology).

### Statistical analysis

All statistical analyses were performed using GraphPad Prism software (version 9.0.0; CA, USA). Differences between two groups were assessed using an unpaired Student’s t-test, while comparisons among multiple groups were conducted using one-way analysis of variance (ANOVA) followed by Tukey’s multiple comparisons test. For experiments involving two independent variables, two-way ANOVA followed by Tukey’s multiple comparisons test was used. For time-course and dose-response experiments, repeated-measures ANOVA was used. Data are expressed as the mean ± standard error of the mean (SEM). A *p*-value of less than 0.05 was considered statistically significant. The levels of statistical significance are denoted as follows: *p* ≥ 0.05 (not significant, NS), *p* < 0.05 (*), *p* < 0.01 (**), *p* < 0.001 (***), and *p* < 0.0001 (****). All experiments were independently repeated at least three times.

## Results

### Ritonavir as a potential binding ligand of IE1

To identify potential IE1-targeting compounds, we performed virtual screening of 890 FDA-approved drugs against the resolved central core domain of IE1 (PDB: 6TGZ) using Discovery Studio 2019 (Fig 1A and S1 Table). Six compounds with LibDock scores >150 were selected, including ritonavir, deferoxamine, atazanavir, lopinavir, salmeterol, and montelukast (S2 Table). Ritonavir showed the highest LibDock score (174.651).

**Fig 1:**
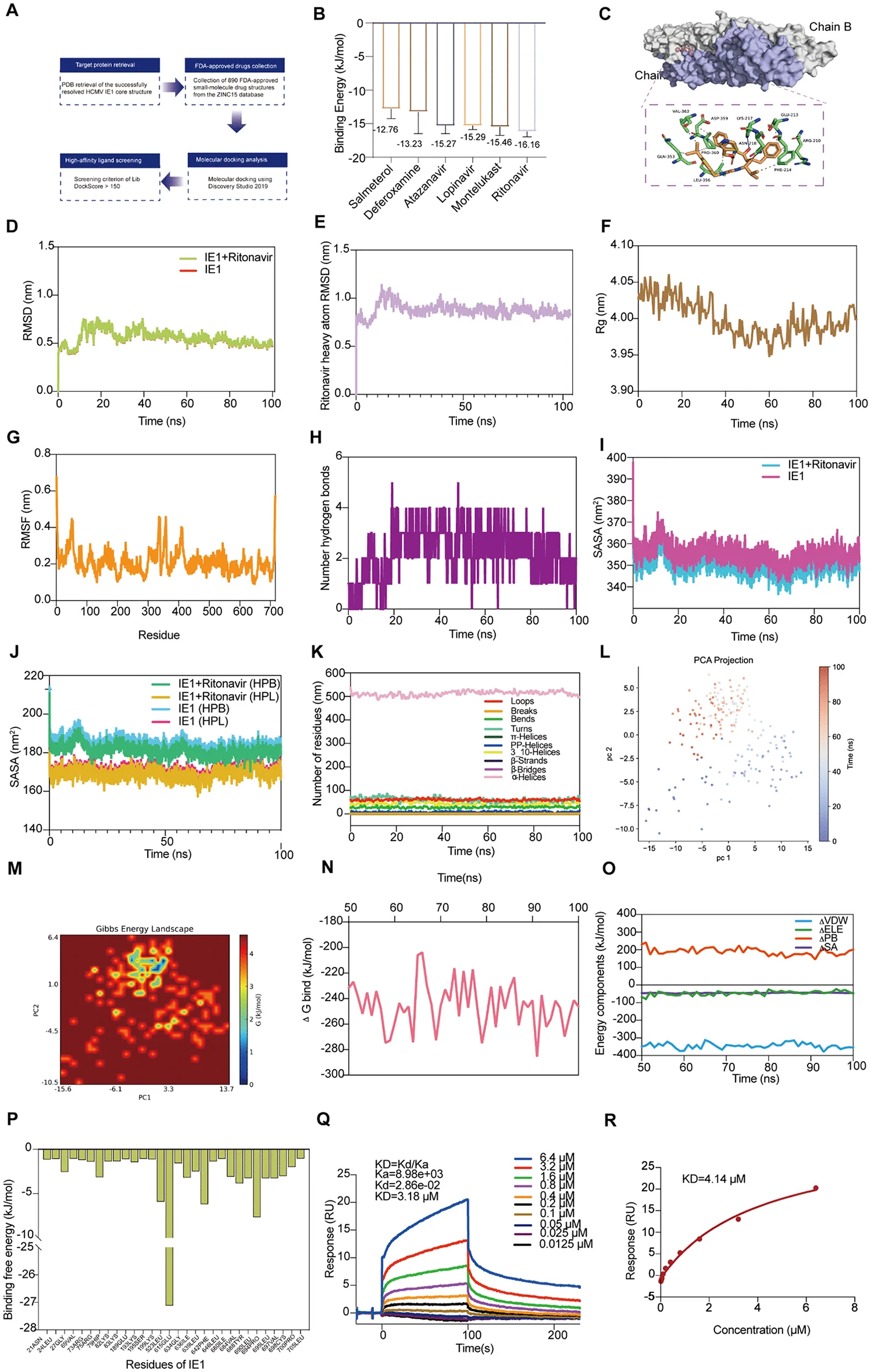
Ritonavir directly binds IE1, as predicted by molecular docking and molecular dynamics simulations and validated by SPR. **(A)** Schematic workflow of the virtual-screening strategy used to identify FDA-approved compounds targeting IE1. A total of 890 compounds were subjected to high-throughput docking using Discovery Studio. **(B)** Predicted binding energies of the six top-ranked candidate compounds with the IE1 core domain, calculated using AutoDock. **(C)** Representative binding pose of ritonavir within the predicted ligand-binding pocket of IE1. **(D–E)** RMSD profiles during 100 ns molecular dynamics simulations. (D) Backbone RMSD of IE1 for the apo and ritonavir-bound systems. (E) RMSD of ritonavir heavy atoms in the IE1-ritonavir complex. **(F–H)** Time-dependent changes in the radius of gyration (Rg) of the IE1-ritonavir complex, residue-level RMSF of ritonavir-bound IE1, and the number of intermolecular hydrogen bonds formed between IE1 and ritonavir during the simulations. **(I–J)** Total solvent-accessible surface area (SASA) of apo-IE1 and the IE1–ritonavir complex, together with time-dependent changes in hydrophobic (HPB) and hydrophilic (HPL) SASA components in both systems. **(K)** Time-dependent changes in secondary-structure residue counts of the IE1–ritonavir complex during the simulations. **(L)** Principal component analysis (PCA) of the molecular dynamics trajectories projected onto the PC1–PC2 plane, with trajectories colored according to simulation time. **(M)** Two-dimensional free-energy landscape constructed from the first two principal components. **(N)** Time-dependent profile of the total MM-PBSA binding free energy of the IE1–ritonavir complex. **(O)** Time-dependent contributions of individual energy components to the calculated binding free energy. ΔVDW, van der Waals energy; ΔELE, electrostatic energy; ΔPB, polar solvation free energy; ΔSA, non-polar solvation free energy. Units: kJ/mol. **(P)** Per-residue energy decomposition of IE1 residues at the predicted ritonavir-binding interface. The y-axis is broken by a double slash (//) to facilitate visualization of residues with smaller energetic contributions. **(Q)** Representative SPR sensorgrams obtained by injecting serial dilutions of ritonavir over immobilized recombinant IE1. Sensorgrams were globally fitted using a 1:1 Langmuir binding model to determine the association rate constant (*k*_*a*_), dissociation rate constant (*k*_*d*_), and equilibrium dissociation constant (*K*_*D*_). **(R)** Steady-state binding curve generated from equilibrium SPR responses at increasing ritonavir concentrations, with the *K*_*D*_ determined by steady-state fitting.

To further prioritize these candidates, potential ligand-binding pockets on the IE1 homodimer were predicted using CB-Dock2, and five candidate pockets were identified (S3 Table). Pocket 1, located at the A/B-subunit interface, was selected for subsequent docking. AutoDock analysis showed that ritonavir had the most favorable predicted binding energy among the six candidates (−16.16 kcal/mol; Fig 1B and 1C). Because ritonavir ranked first in both docking analyses, it was selected for subsequent computational and experimental validation.

The dynamic behavior of the predicted IE1–ritonavir complex was further evaluated by 100 ns molecular dynamics simulations. The complex remained structurally stable during the equilibrated portion of the simulations, with no major perturbation of the global IE1 structure, as supported by RMSD, Rg, RMSF, hydrogen-bond, SASA, secondary-structure, and PCA analyses (Fig 1D–1M). MM-PBSA analysis of the 50–100 ns trajectories further suggested energetically favorable binding, with van der Waals interactions providing a major contribution. Residue-level energy decomposition identified GLU615, LEU523, PHE642, and PRO694 as potential hotspot residues for the predicted interaction (Fig 1N–1P).

To experimentally assess direct binding, SPR assays were performed using purified IE1 protein. Ritonavir exhibited concentration-dependent and reversible binding to immobilized IE1. Kinetic fitting using a 1:1 Langmuir model yielded a *K*_*D*_ of 3.18 μM, while steady-state analysis yielded a comparable *K*_*D*_ of 4.14 μM (Fig 1Q and 1R). These results provide direct biophysical evidence for IE1–ritonavir binding, whereas the docking and molecular dynamics analyses suggest a potential binding mode and candidate interaction residues.

### Ritonavir inhibits IE1 protein expression

Given the predicted interaction between ritonavir and IE1, we first examined whether ritonavir affects IE1 protein abundance. Western blot analysis showed that ritonavir reduced IE1 levels in AD169-infected HFF cells in a time- and dose-dependent manner, with a detectable decrease from day 2 post-infection (Fig 2A and 2B). A similar reduction was observed in HeLa cells stably expressing IE1, indicating that this effect was not dependent on the viral replication context (Fig 2C and 2D). Immunofluorescence analysis further confirmed decreased nuclear IE1 signals in infected HFF cells following ritonavir treatment (Fig 2E and 2F).

**Fig 2:**
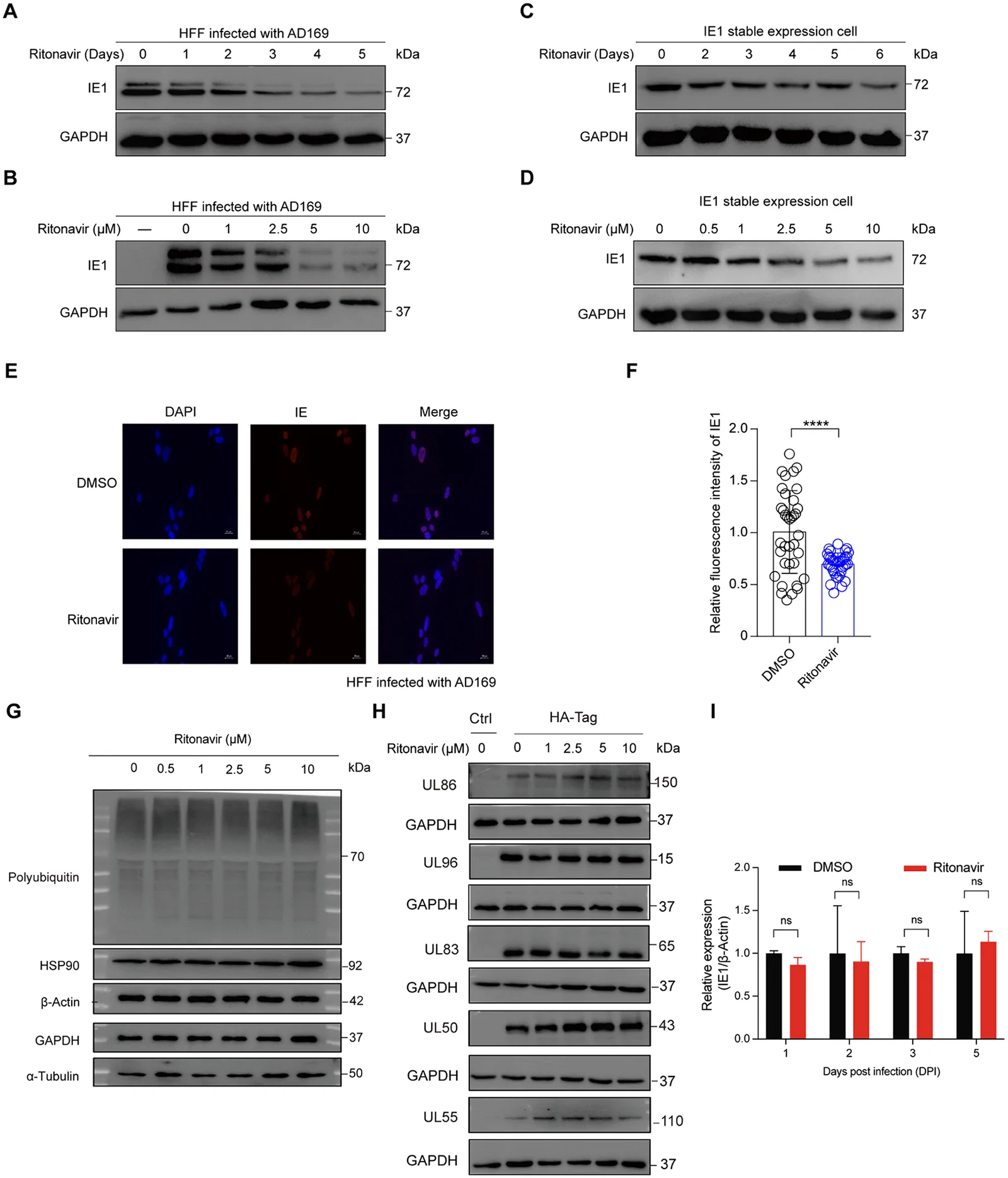
Ritonavir inhibits IE1 protein expression. **(A-B)** HFF cells infected with AD169 were treated with 5 μM Ritonavir for 1–5 days, or with 1–10 μM Ritonavir for 5 days, followed by Western blot analysis of IE1. **(C-D)** IE1 stable cells were treated with 5 μM Ritonavir for 1–5 days, or with 1–10 μM Ritonavir for 5 days, followed by Western blot analysis of IE1. **(E)** HFF cells infected with AD169 were treated with 5 μM Ritonavir or DMSO for 5 days, and then subjected to immunofluorescence staining for IE1. Cell nuclei were labeled with DAPI (blue), and IE1 was detected using an anti-IE1 antibody (red). Scale bar, 20 μm. **(F)** Quantitative fluorescence analysis of IE1 intensity corresponding to panel E. **(G)** HFF cells were treated with increasing concentrations of ritonavir. Western blot analysis was performed to assess the levels of the host proteins GAPDH, β-actin, α-tubulin, and HSP90, as well as global protein ubiquitination. **(H)** HeLa cells were transiently transfected with HA-tagged expression plasmids encoding UL96, UL55, UL86, UL50, or UL83. Following treatment with increasing concentrations of ritonavir for 5 days, the levels of these viral proteins were analyzed by Western blot. **(I)** AD169-infected HFF cells were treated with ritonavir or DMSO, and samples were collected on days 1, 2, 3, and 5 for RT-qPCR analysis of IE1 mRNA levels. Data are presented as mean ± SEM. Statistical significance was assessed by two-way ANOVA followed by Tukey’s multiple comparisons test. NS, not significant; ****p < 0.0001.

To assess the selectivity of this effect, we examined several host proteins and representative HCMV proteins. Ritonavir did not markedly alter the abundance of GAPDH, β-actin, α-tubulin, or HSP90, nor did it induce a global increase in cellular ubiquitination. In addition, the levels of ectopically expressed HCMV proteins UL96, UL55, UL86, UL50, and UL83 were not appreciably reduced (Fig 2G and 2H). These findings suggest a preferential effect of ritonavir on IE1 among the proteins examined.

RT-qPCR analysis further showed that ritonavir did not significantly alter IE1 mRNA levels from days 1–5 post-infection (Fig 2I), suggesting that ritonavir regulates IE1 primarily at the post-transcriptional level.

### Ritonavir facilitates proteasome-mediated degradation of IE1

To investigate the mechanism by which ritonavir affects IE1 protein expression, a cycloheximide (CHX) assay was performed in IE1 stable expression cell. Western blot and subsequent quantification suggested that ritonavir markedly shortened the IE1 half-life upon inhibition of de novo protein synthesis, leading to approximately 40% greater degradation within 9 hours relative to the control (Fig 3A and 3B). These results implied that ritonavir accelerates IE1 degradation.

**Fig 3:**
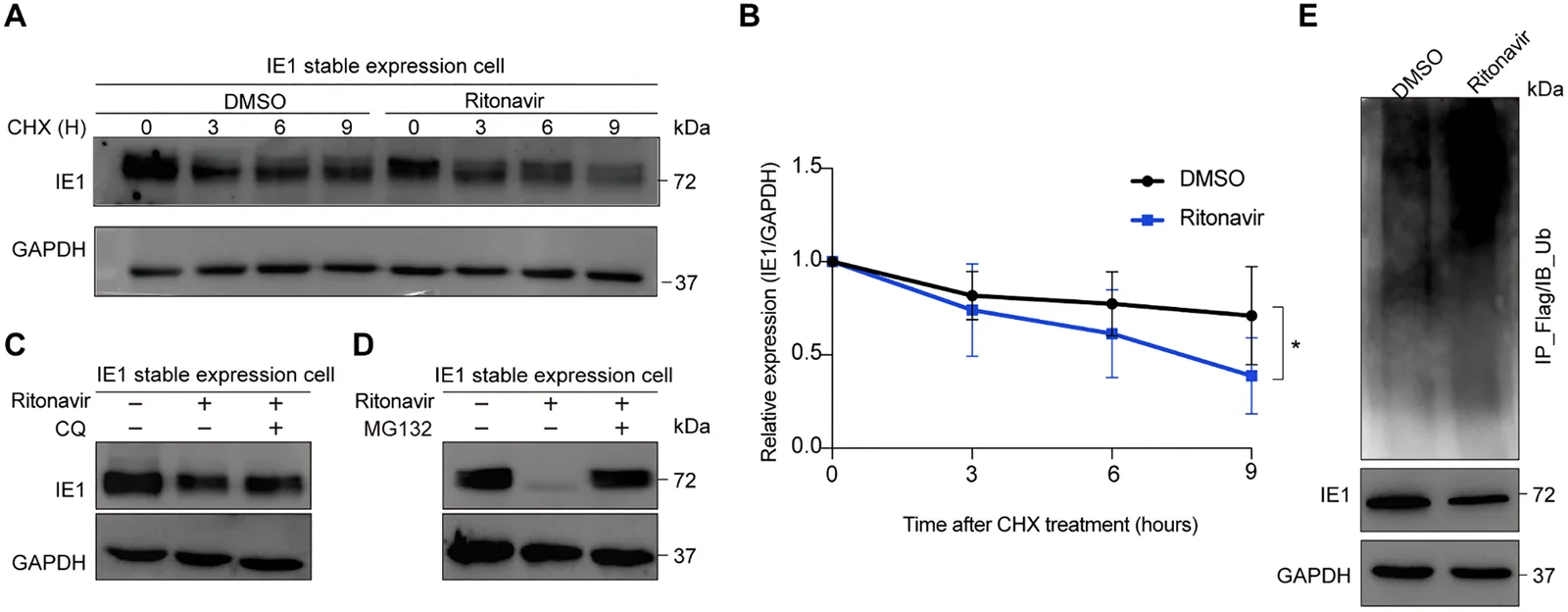
Ritonavir induces IE1 degradation through the ubiquitin-proteasome pathway. **(A)** Western blot analysis of IE1 protein levels in IE1 stable cells treated with CHX (200 μM) in combination with either ritonavir or DMSO for the indicated time points. **(B)** Quantification of IE1 degradation kinetics following CHX treatment in the presence of ritonavir or DMSO at 3, 6, and 9 hours. **(C-D)** Western blot assessment of the effects of the autophagy inhibitor chloroquine (CQ, 50 μM) (C) and the proteasome inhibitor MG132 (10 μM) (D) on ritonavir-induced IE1 degradation. **(E)** Co-immunoprecipitation assay detecting IE1 ubiquitination in Flag-IE1 stably expressing Hela cells treated with ritonavir or DMSO. Flag-tagged IE1 was immunoprecipitated using anti-Flag antibody and ubiquitinated IE1 was probed with anti-ubiquitin antibody. Data are presented as mean ± SEM from three independent biological replicates. Statistical significance was determined by two-way ANOVA followed by Tukey’s multiple comparisons test. *p < 0.05.

Since intracellular proteins are mainly degraded through the ubiquitin-proteasome or autophagy-lysosome pathways28, rescue experiments were conducted using the autophagy inhibitor chloroquine (CQ) and the proteasome inhibitor MG132 to elucidate how ritonavir promotes IE1 degradation (Fig 3C and 3D). Western blot analysis confirmed that MG132 partially reversed ritonavir-induced IE1 degradation (Fig 3D), whereas CQ had no effect (Fig 3C), suggesting that ritonavir promotes IE1 degradation via the ubiquitin-proteasome system. Given that ubiquitination signals proteasomal degradation, co-immunoprecipitation assays were performed to assess IE1 ubiquitination. After immunoprecipitation with anti-Flag antibody, ubiquitinated IE1 was detected with an anti-ubiquitin antibody. As presented in Fig 3E, ritonavir treatment notably increased IE1 ubiquitination. As a whole, these results demonstrate that ritonavir enhances IE1 ubiquitination and activates the ubiquitin-proteasome pathway, thereby promoting IE1 degradation.

### Ritonavir exhibits potent anti-HCMV activity

Given that ritonavir regulates IE1 protein expression, we further investigated whether it affects HCMV proliferation. We conducted a series of *in vitro* antiviral assays (Fig 4A). First, the cytotoxicity of ritonavir was evaluated using flow cytometry and CCK-8 assays. The results showed that at specific concentrations, ritonavir had no significant effect on host cell apoptosis or viability (Fig 4B–4D). On this basis, we examined the antiviral efficacy of ritonavir. The results demonstrated that compared with the DMSO control group (31.52 ± 4.51 copies/mL), ritonavir treatment significantly reduced viral DNA loads in the supernatant of infected cells to 11.75 ± 2.50 copies/mL, representing an inhibition rate of approximately 62.7% (Fig 4E). TCID_50_ assays further confirmed that ritonavir significantly reduced the production of infectious viral particles in a dose-dependent manner (Fig 4F). We subsequently validated these findings using a recombinant HCMV expressing GFP (rHCMV). Fresh HFF cells were reinfected with the supernatant collected after ritonavir treatment, and GFP fluorescence intensity was used as an indicator of infectious viral particle levels. Both fluorescence imaging and quantitative analysis showed that ritonavir treatment reduced GFP signals by approximately 2.2-fold (Fig 4G–4H). Finally, we examined the expression dynamics of key viral proteins in AD169-infected HFF cells. Western blot results revealed that with prolonged ritonavir treatment, the expression levels of IE1, UL44, UL83, and UL84 were all significantly suppressed (Fig 4I). Collectively, these results confirm that ritonavir possesses anti-HCMV activity.

**Fig 4:**
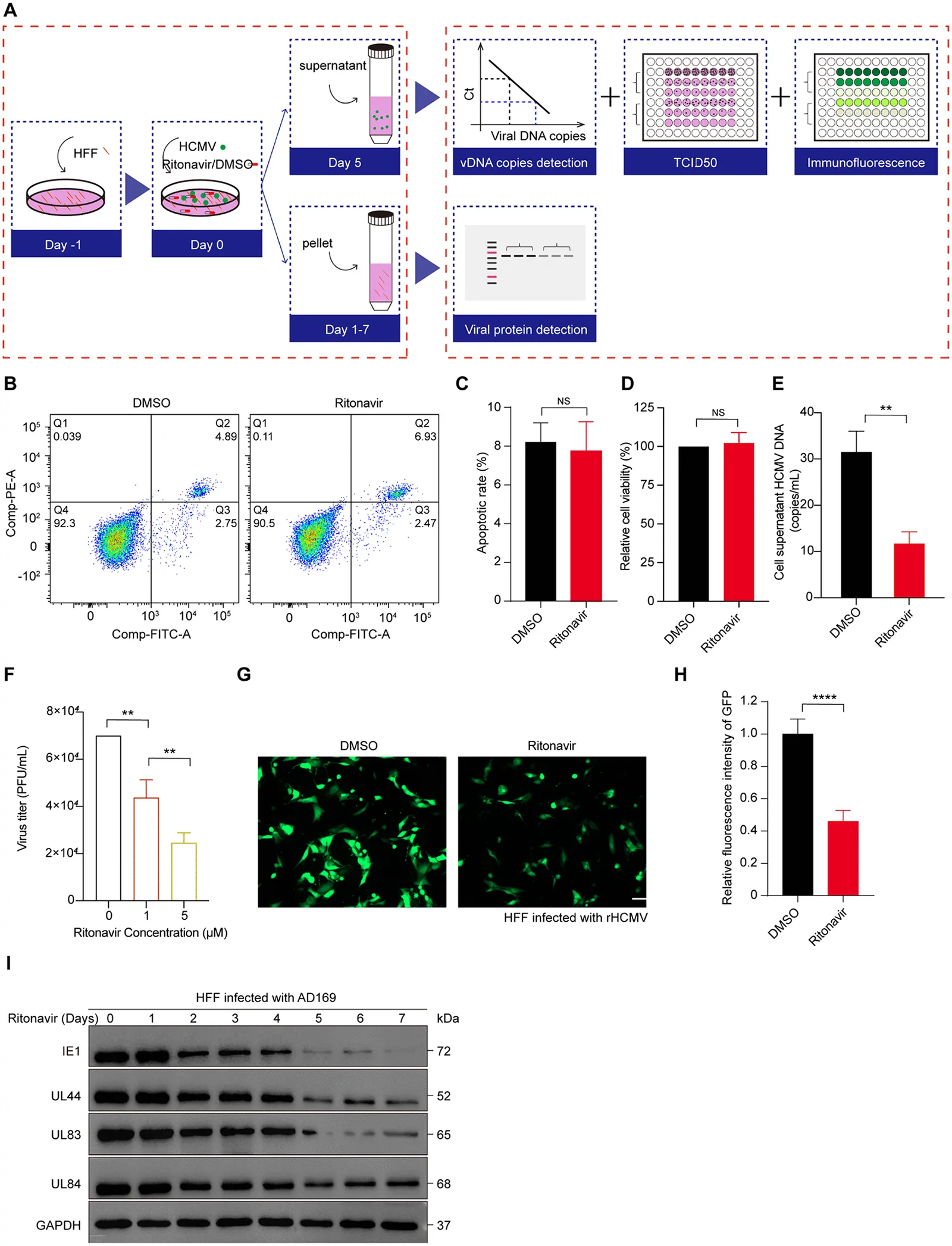
Functional validation of ritonavir-mediated inhibition of HCMV replication. **(A)** Schematic workflow of the virus replication experiment. **(B-C)** Flow cytometry analysis of apoptosis levels in HFF cells following treatment with 5 μM ritonavir for 5 days, with quantitative results shown. **(D)** Cytotoxicity evaluation of HFF cells treated with 5 μM ritonavir for 5 days using the CCK-8 assay. **(E)** HFF cells were infected with AD169 and treated with 5 μM ritonavir for 5 days. Supernatants were collected, DNA was extracted, and qPCR analysis demonstrated that ritonavir significantly reduced progeny virus production of AD169. **(F)** HFF cells were treated with 1 or 5 μM ritonavir for 5 days, and viral titers in the culture supernatants were determined using the TCID_50_ assay. **(G)** Antiviral activity of ritonavir was assessed using an HCMV-based GFP reporter system. Supernatants from rHCMV infected cells treated with 5 μM ritonavir for 5 days were used to re-infect fresh HFF cells for 96 hours. Representative fluorescence images are shown. **(H)** Quantitative fluorescence analysis of GFP intensity corresponding to panel G. **(I)** Western blot analysis of viral proteins (IE1, UL44, UL83, and UL84) in AD169-infected HFF cells treated with 5 μM ritonavir for 1 to 7 days. Scale bar: 20 μm. Data are presented as mean ± SEM from a representative experiment performed in triplicate. Statistical analyses were performed using either one-way ANOVA with Tukey’s post hoc test or unpaired two-tailed Student’s t-test, as indicated in figure legends. NS, not significant; *p < 0.05, **p < 0.01, ****p < 0.0001.

### Ritonavir and ganciclovir synergistically inhibit HCMV replication

GCV inhibits viral DNA polymerase, whereas LMV targets the viral terminase complex to block genome cleavage and packaging (Fig 5A). We first evaluated the cytotoxicity and antiviral activity of ritonavir, GCV, and LMV in HFF cells. All three drugs inhibited HCMV replication in a dose-dependent manner. Ritonavir showed a CC_50_ of 57.36 ± 6.57 μM and an EC_50_ of 6.10 μM (95% CI, 4.31–11.27 μM), corresponding to a selectivity index of 9.40. GCV showed minimal cytotoxicity within the tested range (CC_50_ > 128 μM; EC_50_, 1.80 μM), whereas LMV showed a CC_50_ of 61.06 ± 7.53 μM and an EC_50_ of 5.26 nM (Fig 5B–5G).

**Fig 5:**
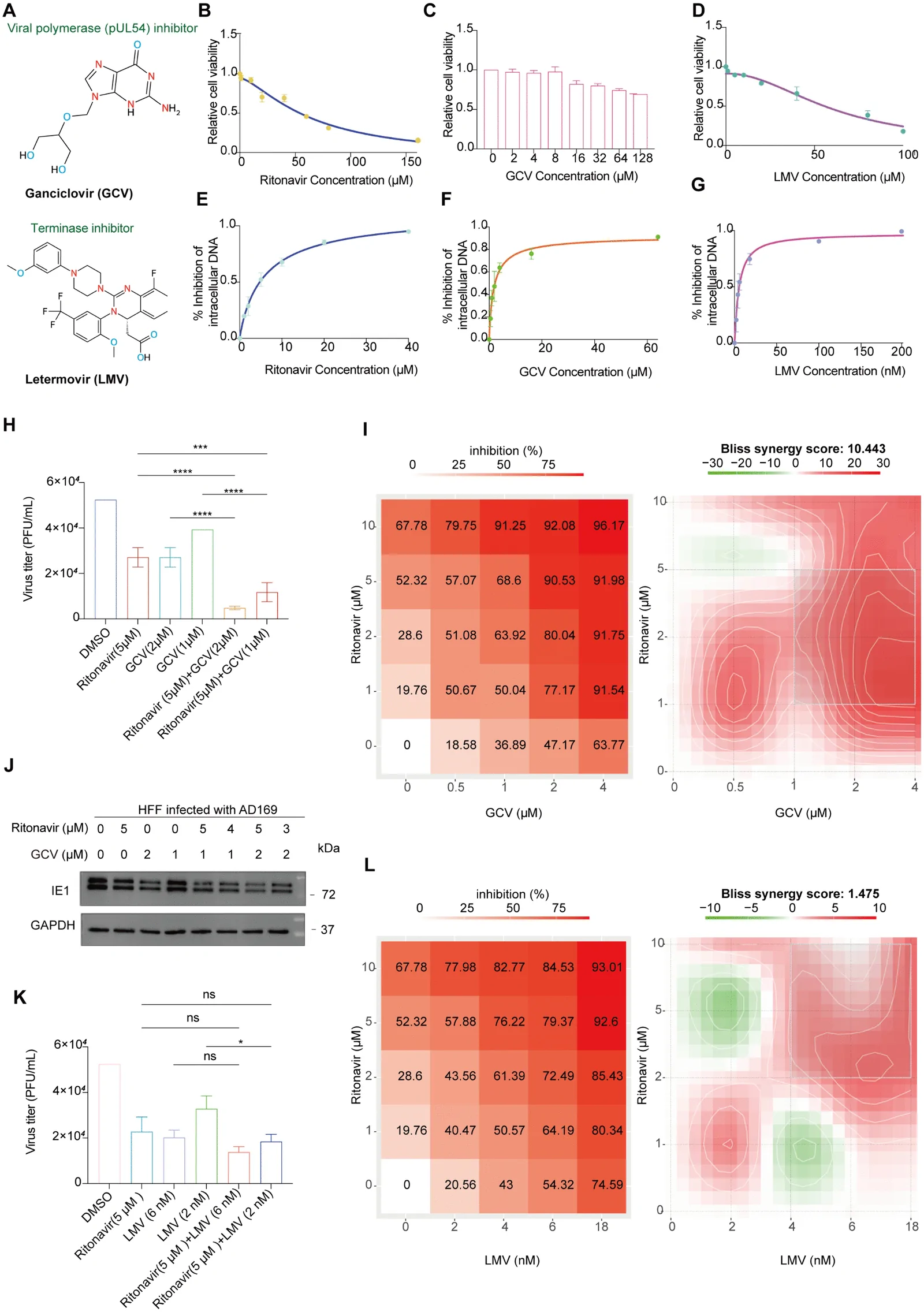
Ritonavir and ganciclovir synergistically inhibit HCMV replication. **(A)** Chemical structures of GCV and LMV. **(B-D)** HFF cells were treated with serially diluted ritonavir, GCV, or LMV for 5 days. Cell viability was measured by the CCK-8 assay. CC₅₀ values were obtained via curve fitting (B, D). Bar graph showing cell viability across GCV concentrations (C). **(E-G)** Following HCMV-AD169 infection, HFF cells were treated with serially diluted compounds for 5 days. Intracellular viral DNA loads were quantified by qPCR, and viral inhibition rates were calculated to determine EC_50_ values. **(H)** TCID_50_ assay was performed to evaluate the interaction between ritonavir and GCV against HCMV. **(I)** Combination matrix constructed under the Bliss independence model to assess anti-HCMV activity for ritonavir plus GCV. Left: percentage inhibition matrix; Right: corresponding Bliss synergy-score matrix. Bliss independent prediction: *E*_*bliss*_ = *E*_*a*_ + *E*_*b*_ – *E*_*a*_*E*_*b*_. Bliss Score = *E*_*observed*_ – *E*_*bliss*_, Bliss synergy scores were computed using SynergyFinder (https://synergyfinder.fimm.fi). A score > 10 defines synergistic interaction, scores between −10 and 10 indicate additive effects, and a score <−10 denotes antagonism. The square box indicates the region of the most synergistic area score. **(J)** Western blot results show the inhibitory effect of the combined treatment of ritonavir and GCV on the IE1 protein. **(K)** TCID_50_ assay was performed to evaluate the interaction between ritonavir and LMV against HCMV. **(L)** Combination matrix constructed under the Bliss independence model to assess anti-HCMV activity for ritonavir plus LMV. Left: percentage inhibition matrix; Right: corresponding Bliss synergy-score matrix. Data are presented as mean ± SEM from a representative experiment performed in triplicate. Statistical analysis was performed using one-way ANOVA with Tukey’s post hoc test. NS, not significant; *p < 0.05, **p < 0.01, ***p < 0.001, ****p < 0.0001.

We next assessed the antiviral effects of ritonavir combined with GCV or LMV using TCID_50_ assays and Bliss independence analysis. Ritonavir plus GCV showed reproducible synergy. For example, 5 μM ritonavir combined with 2 μM GCV achieved 90.83% inhibition, compared with a Bliss-predicted value of 73.43%. Consistently, 5 × 5 checkerboard analysis yielded an overall Bliss synergy score of 10.443 and a most synergistic area score of 12.10 (Fig 5H and 5I). The combination also reduced IE1 protein levels more markedly than either drug alone, consistent with the enhanced antiviral effect of the combination (Fig 5J).

In contrast, ritonavir plus LMV did not show meaningful synergy. The observed antiviral effects at representative dose combinations were lower than the Bliss-predicted values, and 5 × 5 matrix analysis yielded an overall synergy score of 1.475 and a most synergistic area score of 2.79 (Fig 5K and 5L). Together, these results indicate that ritonavir synergizes with GCV, but not with LMV, against HCMV.

## Discussion

Ritonavir, a well-established HIV protease inhibitor [31], primarily functions as a pharmacokinetic booster in combinations such as Paxlovid by inhibiting CYP3A4 [32], thereby decreasing the metabolism and clearance of co-administered drugs [33,34]. Through molecular docking and *in vitro* experiments (Figs 1 and 4), our study uncovers for the first time that ritonavir possesses a distinct antiviral activity against HCMV.

Conventional anti-HCMV drugs mainly inhibit enzymes required for viral DNA replication [7,8], whereas this work provides the validation of IE1 as an effective and feasible target. IE1 is a key regulator that promotes viral replication by interacting with ND10 components (PML, Sp100, and Daxx) and the ISGF3 complex (STAT2) [35–38], thereby counteracting host intrinsic restriction and innate immune signaling. Loss of IE1 function redirects infection toward a host-favorable antiviral state (Fig 6). Despite the strong species specificity of HCMV, comparative analyses uncover that the core domain of IE1 is highly conserved among humans, rhesus macaques, and rats [25,39], highlighting that ritonavir targeting of IE1 could enable a cross-species, broad-spectrum antiviral approach. Mechanistic studies suggest that ritonavir inhibits HCMV replication by promoting ubiquitin-proteasome-mediated degradation of IE1 (Figs 2 and 3). Unlike conventional viral enzyme inhibitors, this mechanism suggests that pharmacological destabilization of key viral proteins may represent an alternative antiviral strategy. Although ritonavir itself is neither a PROTAC nor a molecular-glue degrader, its mode of action is conceptually related to targeted protein degradation [40–44].

**Fig 6:**
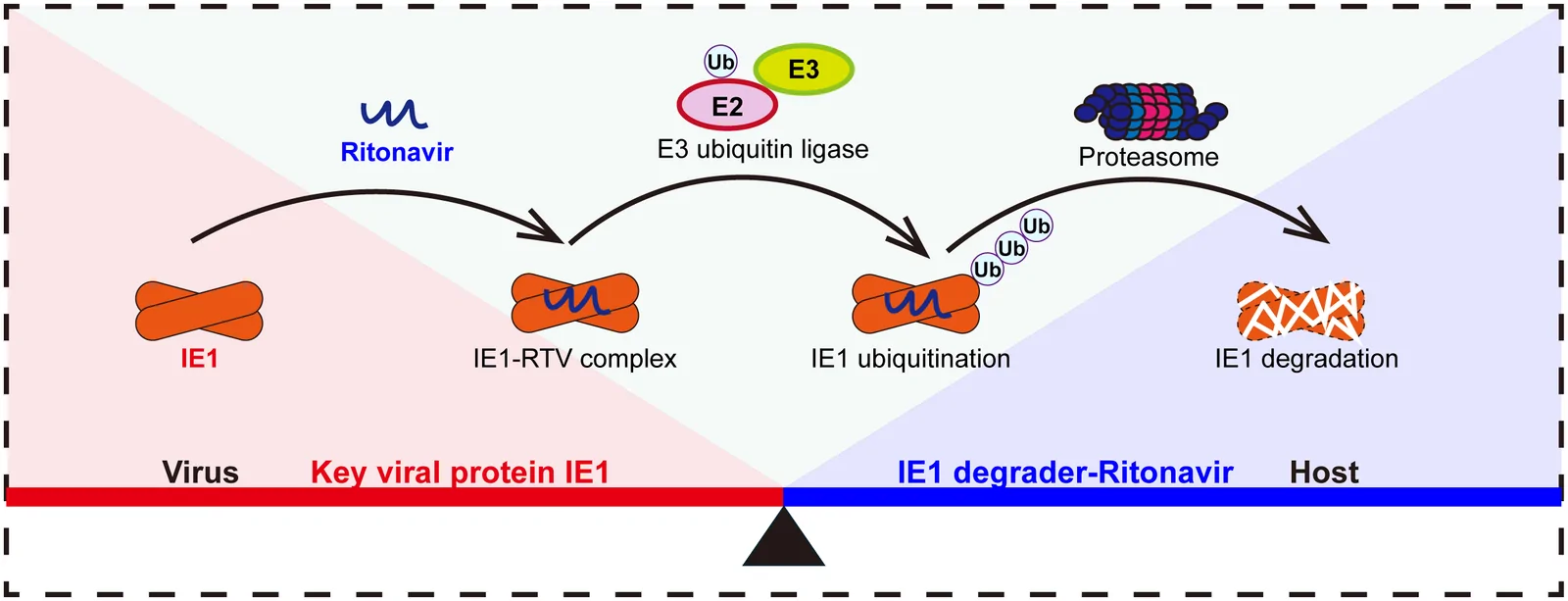
Schematic summary of the proposed mechanism. Ritonavir, identified through molecular docking screening, binds to IE1 and promotes its ubiquitination-dependent degradation, thereby exerting anti-HCMV activity.

Notably, ritonavir exhibited anti-HCMV activity *in vitro* and showed synergistic effects with GCV, whereas no obvious synergy was observed with LMV (Fig 5). Ritonavir promotes IE1 degradation and affects early viral regulation, while GCV inhibits viral DNA polymerase. These distinct mechanisms may contribute to their synergistic activity. In contrast, the basis for the lack of synergy between ritonavir and LMV remains to be clarified.

In addition, HIV and HCMV co-infection is common in immunocompromised patients. Previous studies have shown that some HIV inhibitors can directly suppress herpesvirus replication in addition to their effects through immune restoration [45,46]. Our findings further demonstrate that ritonavir has direct anti-HCMV activity, supporting its potential for drug repurposing and combination therapy with GCV.

Several limitations remain in the present study. First, our drug-screening pipeline relies on conventional LibDock and AutoDock molecular docking for candidate prioritization, without adopting emerging AI-driven drug-discovery frameworks such as network-based repurposing (DRHIN), diffusion-model docking (SurfDock), equivariant deep-learning pose prediction (RAPiDock), and quantum-chemistry-integrated LiTENexus [47–50]. Compared with these advanced AI tools, our workflow lacks retrospective benchmarking using decoy-set enrichment and ROC-AUC evaluation, which prevents comprehensive quantification of model discriminatory performance.

Mechanistically, ritonavir promoted IE1 ubiquitination and proteasome-associated degradation, but MG132 only partially rescued the reduction in IE1, and the responsible E3 ubiquitin ligase remains unknown. In addition, binding-site mutagenesis and IE1 rescue experiments are still lacking, preventing definitive establishment of a direct causal link between IE1 degradation and the anti-HCMV effect.

Finally, most experiments were performed in HFF and HeLa cells using the laboratory-adapted HCMV AD169 strain, without validation in clinical isolates, drug-resistant strains, or *in vivo* models. Ritonavir showed a selectivity index of approximately 9.40, indicating moderate *in vitro* selectivity. Whether the effective concentrations are clinically achievable and whether the synergy with GCV can be reproduced *in vivo* remain to be determined.

In summary, this study identifies for the first time that ritonavir is an inhibitor that regulates IE1 protein expression. This finding provides a promising therapeutic option and combination strategy for drug-resistant HCMV infection.

## Supporting information

S1 Table Ranking of docking scores for 890 small molecules. (XLSX)

S2 Table Potential high-affinity ligands identified from molecular docking. (DOCX)

S3 Table Prediction of potential small-molecule binding pockets on the IE1 protein. (DOCX)

S4 Table Raw viral titers, percentage inhibition, and Bliss-independent synergy analysis for drug combination assays. (DOCX)
