# Supplementary material for: Repurposing ritonavir to induce proteasomal degradation of IE1 for inhibition of human cytomegalovirus replication
Source: PLoS Comput Biol. 2026 Oct 6;22(10):e1014867. doi: 10.1371/journal.pcbi.1014867 (PMC13705602; doi:10.1371/journal.pcbi.1014867)
Supplement: Supplementary file 1 [file pcbi.1014867.s002.docx]

**S2 Table. Potential high-affinity ligands identified from molecular docking.**

|  | ZINC-ID | Name | LibDock score |
| --- | --- | --- | --- |
| 1 | ZINC000003944422 | Ritonavir | 174.651 |
| 2 | ZINC000003830635 | Deferoxamine | 169.712 |
| 3 | ZINC000003941496 | Atazanavir | 164.758 |
| 4 | ZINC000003951740 | Lopinavir | 156.545 |
| 5 | ZINC000003799072 | Salmeterol | 153.916 |
| 6 | ZINC000003831151 | Montelukast | 153.445 |
