# Supplementary material for: Repurposing ritonavir to induce proteasomal degradation of IE1 for inhibition of human cytomegalovirus replication
Source: PLoS Comput Biol. 2026 Oct 6;22(10):e1014867. doi: 10.1371/journal.pcbi.1014867 (PMC13705602; doi:10.1371/journal.pcbi.1014867)
Supplement: Supplementary file 2 [file pcbi.1014867.s003.docx]

**S3 Table. Prediction of potential small-molecule binding pockets on the IE1 protein**

| Pocket ID | Cavity volume（Å³） | | Center coordinate  (x, y, z) | Grid dimension(Å) | Druggability score | |
| --- | --- | --- | --- | --- | --- | --- |
| Pocket 1 | 5036 | 11.010, −15.048, 0.547 | | 27 × 27 × 35 | | −8.0 |
| Pocket 2 | 1953 | −11.176, −9.655,39.139 | | 27 × 27 × 33 | | −8.0 |
| Pocket 3 | 1842 | −24.651,−12.905,57.098 | | 27 × 27 × 27 | | −6.4 |
| Pocket 4 | 890 | −0.689, −14.417, 17.521 | | 27 × 27 × 27 | | −7.1 |
| Pocket 5 | 464 | −12.217,−21.154,35.271 | | 27 × 27 × 27 | | −8.0 |
