# Supplementary material for: Repurposing ritonavir to induce proteasomal degradation of IE1 for inhibition of human cytomegalovirus replication
Source: PLoS Comput Biol. 2026 Oct 6;22(10):e1014867. doi: 10.1371/journal.pcbi.1014867 (PMC13705602; doi:10.1371/journal.pcbi.1014867)
Supplement: Supplementary file 3 [file pcbi.1014867.s004.docx]

**Figure5 H**

| Raw Viral titers | | | |
| --- | --- | --- | --- |
| Condition | Rep1 | Rep2 | Rep3 |
| DMSO (Negative Control) | 52492.59 | 52492.59 | 52492.59 |
| Ritonavir(5 μM) | 22135.94 | 29518.76 | 29518.76 |
| GCV(1 μM) | 39363.89 | 39363.89 | 39363.89 |
| GCV(2 μM) | 29518.76 | 22135.94 | 29518.76 |
| Ritonavir + GCV(5 μM + 1 μM) | 9334.65 | 16599.62 | 9334.65 |
| Ritonavir + GCV(5 μM + 2 μM) | 3936.39 | 5249.26 | 5249.26 |

| Percentage Inhibition (%) | | | | | |
| --- | --- | --- | --- | --- | --- |
| Condition | Rep1 (%) | Rep2 (%) | Rep3 (%) | Mean (%) | SD (%) |
| DMSO (Negative Control) | 0 | 0 | 0 | 0 | 0 |
| Ritonavir(5 μM) | 57.83 | 43.77 | 43.77 | 48.45 | 8.12 |
| GCV(1 μM) | 25.01 | 25.01 | 25.01 | 25.01 | 0 |
| GCV(2 μM) | 43.77 | 57.83 | 43.77 | 48.45 | 8.12 |
| Ritonavir + GCV(5 μM + 1 μM) | 82.22 | 68.38 | 82.22 | 77.6 | 7.99 |
| Ritonavir + GCV(5 μM + 2 μM) | 92.5 | 90 | 90 | 90.83 | 1.44 |

| 联用组合 | EA (%) | EB (%) | Expected (%) | Observed (%) | Bliss excess (%) |
| --- | --- | --- | --- | --- | --- |
| Ritonavir+GCV(5+1) | 48.45 | 25.01 | **61.34** | 77.60 | **16.26** |
| Ritonavir+GCV(5+2) | 48.45 | 48.45 | **73.43** | 90.83 | **17.40** |

| Test details | Mean 1 | | Mean 2 | | Mean Diff. | | SE of diff. | | n1 | 95.00% CI of diff. |
| --- | --- | --- | --- | --- | --- | --- | --- | --- | --- | --- |
| DMSO vs. Ritonavir(5μM) | 52493 | 27058 | | 25435 | | 2461 | | 3 | | 17169 to 33701 |
| DMSO vs. Ritonavir (5μM)+GCV(2μM) | 52493 | 4812 | | 47681 | | 2461 | | 3 | | 39415 to 55947 |
| DMSO vs. GCV(2μM) | 52493 | 27058 | | 25435 | | 2461 | | 3 | | 17169 to 33701 |
| DMSO vs. GCV(1μM) | 52493 | 39364 | | 13129 | | 2461 | | 3 | | 4863 to 21395 |
| DMSO vs. Ritonavir(5μM)+GCV(1μM) | 52493 | 11756 | | 40736 | | 2461 | | 3 | | 32470 to 49002 |
| Ritonavir(5μM) vs. Ritonavir (5μM)+GCV(2μM) | 27058 | 4812 | | 22246 | | 2461 | | 3 | | 13980 to 30512 |
| Ritonavir(5μM) vs. GCV(2μM) | 27058 | 27058 | | 0 | | 2461 | | 3 | | -8266 to 8266 |
| Ritonavir(5μM) vs. GCV(1μM) | 27058 | 39364 | | -12306 | | 2461 | | 3 | | -20572 to -4040 |
| Ritonavir(5μM) vs. Ritonavir(5μM)+GCV(1μM) | 27058 | 11756 | | 15302 | | 2461 | | 3 | | 7035 to 23568 |
| Ritonavir (5μM)+GCV(2μM) vs. GCV(2μM) | 4812 | 27058 | | -22246 | | 2461 | | 3 | | -30512 to -13980 |
| Ritonavir (5μM)+GCV(2μM) vs. GCV(1μM) | 4812 | 39364 | | -34552 | | 2461 | | 3 | | -42818 to -26286 |
| Ritonavir (5μM)+GCV(2μM) vs. Ritonavir(5μM)+GCV(1μM) | 4812 | 11756 | | -6945 | | 2461 | | 3 | | -15211 to 1321 |
| GCV(2μM) vs. GCV(1μM) | 27058 | 39364 | | -12306 | | 2461 | | 3 | | -20572 to -4040 |
| GCV(2μM) vs. Ritonavir(5μM)+GCV(1μM) | 27058 | 11756 | | 15302 | | 2461 | | 3 | | 7035 to 23568 |
| GCV(1μM) vs. Ritonavir(5μM)+GCV(1μM) | 39364 | 11756 | | 27608 | | 2461 | | 3 | | 19342 to 35874 |

**Figure5 I**

| 观察到的抑制率矩阵（Ritonavir × GCV） | | | | | |
| --- | --- | --- | --- | --- | --- |
| Ritonavir \ GCV | 0 μM | 0.5 μM | 1 μM | 2 μM | 4 μM |
| 0 μM | 0 | 18.58 | 36.89 | 47.17 | 63.77 |
| 1 μM | 19.76 | 50.67 | 50.04 | 77.17 | 91.54 |
| 2 μM | 28.6 | 51.08 | 63.92 | 80.04 | 91.75 |
| 5 μM | 52.32 | 57.07 | 68.6 | 90.53 | 91.98 |
| 10 μM | 67.78 | 79.75 | 91.25 | 92.08 | 96.17 |

| Bliss 预测值矩阵（E_bliss_ = E_a_ + E_b_ − E_a_E_b_） | | | | | |
| --- | --- | --- | --- | --- | --- |
| Ritonavir \ GCV | 0 μM | 0.5 μM | 1 μM | 2 μM | 4 μM |
| 0 μM | 0 | 18.58 | 36.89 | 47.17 | 63.77 |
| 1 μM | 19.76 | 34.24 | 49.51 | 57.98 | 72.91 |
| 2 μM | 28.6 | 41.94 | 55.91 | 64.6 | 77.9 |
| 5 μM | 52.32 | 61.21 | 69.89 | 74.81 | 82.74 |
| 10 μM | 67.78 | 74.77 | 81.01 | 84.45 | 89.99 |

| Bliss synergy score 矩阵（Score = E_observed_ − E_bliss_） | | | | | |
| --- | --- | --- | --- | --- | --- |
| Ritonavir \ GCV | 0 μM | 0.5 μM | 1 μM | 2 μM | 4 μM |
| 0 μM | 0 | 0 | 0 | 0 | 0 |
| 1 μM | 0 | 16 | 0.68 | 19.56 | 20.61 |
| 2 μM | 0 | 9.21 | 8.98 | 17.76 | 17.62 |
| 5 μM | 0 | -4.11 | -1.31 | 15.72 | 9.25 |
| 10 μM | 0 | 5.98 | 11.58 | 9.1 | 7.84 |

Figure5 K

| Raw Viral titers | | | |
| --- | --- | --- | --- |
| Condition | Rep1 | Rep2 | Rep3 |
| Virus control (0 μM) | 52492.5947 | 52492.5947 | 52492.5947 |
| LMV (6 μM) | 22135.9436 | 16599.6159 | 22135.9436 |
| LMV6 μM + RIT 5 μM | 12447.9559 | 16599.6159 | 12447.9559 |
| RIT (5 μM) | 29518.7552 | 16599.6159 | 22135.9436 |
| LMV (2 μM) | 29518.7552 | 29518.7552 | 39363.8928 |
| LMV 2 μM + RIT 5 μM | 22135.9436 | 16599.6159 | 16599.6159 |

| Percentage Inhibition (%) | | | | | |
| --- | --- | --- | --- | --- | --- |
| Condition | Rep1 (%) | Rep2 (%) | Rep3 (%) | Mean (%) | SD (%) |
| Virus control (0 μM) | 0 | 0 | 0 | 0 | 0 |
| LMV (6 μM) | 57.83 | 68.38 | 57.83 | 61.35 | 4.97 |
| LMV6 μM + RIT 5 μM | 76.29 | 68.38 | 76.29 | 73.65 | 3.73 |
| RIT (5 μM) | 43.77 | 68.38 | 57.83 | 56.66 | 10.08 |
| LMV (2 μM) | 43.77 | 43.77 | 25.01 | 37.51 | 8.84 |
| LMV 2 μM + RIT 5 μM | 37.51 | 56.66 | 64.86 | 72.92 | -8.06 |

| 联用组合 | EA (%) | EB (%) | Expected (%) | Observed (%) | Bliss excess (%) |
| --- | --- | --- | --- | --- | --- |
| LMV 6 μM + RIT 5 μM | 61.35 | 56.66 | **83.24** | 73.65 | **‑9.59** |
| LMV 2 μM + RIT 5 μM | 37.52 | 56.66 | **73.03** | 64.86 | **‑8.17** |

| Test details | Mean 1 | Mean 2 | Mean Diff. | SE of diff. | n1 | 95.00% CI of diff. |
| --- | --- | --- | --- | --- | --- | --- |
| DMSO vs. LMV (6 nM) | 52493 | 20291 | 32202 | 3342 | 3 | 20978 to 43426 |
| DMSO vs. Ritonavir(5 μM )+LMV (6 nM) | 52493 | 13832 | 38661 | 3342 | 3 | 27436 to 49885 |
| DMSO vs. Ritonavir(5 μM ) | 52493 | 22751 | 29741 | 3342 | 3 | 18517 to 40965 |
| DMSO vs. LMV (2 nM) | 52493 | 32800 | 19692 | 3342 | 3 | 8468 to 30916 |
| DMSO vs. Ritonavir(5 μM )+LMV (2 nM) | 52493 | 18445 | 34048 | 3342 | 3 | 22823 to 45272 |
| LMV (6 nM) vs. Ritonavir(5 μM )+LMV (6 nM) | 20291 | 13832 | 6459 | 3342 | 3 | -4766 to 17683 |
| LMV (6 nM) vs. Ritonavir(5 μM ) | 20291 | 22751 | -2461 | 3342 | 3 | -13685 to 8763 |
| LMV (6 nM) vs. LMV (2 nM) | 20291 | 32800 | -12510 | 3342 | 3 | -23734 to -1286 |
| LMV (6 nM) vs. Ritonavir(5 μM )+LMV (2 nM) | 20291 | 18445 | 1845 | 3342 | 3 | -9379 to 13070 |
| Ritonavir(5 μM )+LMV (6 nM) vs. Ritonavir(5 μM ) | 13832 | 22751 | -8920 | 3342 | 3 | -20144 to 2305 |
| Ritonavir(5 μM )+LMV (6 nM) vs. LMV (2 nM) | 13832 | 32800 | -18969 | 3342 | 3 | -30193 to -7744 |
| Ritonavir(5 μM )+LMV (6 nM) vs. Ritonavir(5 μM )+LMV (2 nM) | 13832 | 18445 | -4613 | 3342 | 3 | -15837 to 6611 |
| Ritonavir(5 μM ) vs. LMV (2 nM) | 22751 | 32800 | -10049 | 3342 | 3 | -21273 to 1175 |
| Ritonavir(5 μM ) vs. Ritonavir(5 μM )+LMV (2 nM) | 22751 | 18445 | 4306 | 3342 | 3 | -6918 to 15531 |

Figure5 L

| 观察到的抑制率矩阵（Ritonavir × LMV） | | | | | |
| --- | --- | --- | --- | --- | --- |
| Ritonavir \ LMV | 0 nM | 2 nM | 4 nM | 6 nM | 18 nM |
| 0 μM | 0 | 20.56 | 43 | 54.32 | 74.59 |
| 1 μM | 19.76 | 40.47 | 49.59 | 64.19 | 80.34 |
| 2 μM | 28.6 | 43.56 | 61.39 | 72.49 | 85.43 |
| 5 μM | 52.32 | 57.88 | 76.22 | 79.37 | 92.6 |
| 10 μM | 67.78 | 77.98 | 82.77 | 84.53 | 93.01 |

| Bliss 预测值矩阵（E_bliss_ = E_a_ + E_b_ − E_a_E_b_） | | | | | |
| --- | --- | --- | --- | --- | --- |
| Ritonavir \ LMV | 0 nM | 2 nM | 4 nM | 6 nM | 18 nM |
| 0 μM | 0 | 20.56 | 43 | 54.32 | 74.59 |
| 1 μM | 19.76 | 36.26 | 54.26 | 63.35 | 79.61 |
| 2 μM | 28.6 | 43.28 | 59.3 | 67.38 | 81.86 |
| 5 μM | 52.32 | 62.12 | 72.82 | 78.22 | 87.88 |
| 10 μM | 67.78 | 74.4 | 81.63 | 85.28 | 91.81 |

| Bliss synergy score 矩阵（Score = E_observed_ − E_bliss_） | | | | | |
| --- | --- | --- | --- | --- | --- |
| Ritonavir \ LMV | 0 nM | 2 nM | 4 nM | 6 nM | 18 nM |
| 0 μM | 0 | 0 | 0 | 0 | 0 |
| 1 μM | 0 | 4.21 | ‑4.67 | 0.84 | 0.73 |
| 2 μM | 0 | 0.28 | 2.09 | 5.11 | 3.57 |
| 5 μM | 0 | ‑4.24 | 3.4 | 1.15 | 4.72 |
| 10 μM | 0 | 3.58 | 1.14 | ‑0.75 | 1.2 |
